# Clinical impact of central nervous system‐directed therapies on intravascular large B‐cell lymphoma: A single institution's experience

**DOI:** 10.1002/jha2.380

**Published:** 2022-02-02

**Authors:** Hiromichi Takahashi, Haruna Nishimaki, Yoko Nakanishi, Takashi Hamada, Masaru Nakagawa, Kazuhide Iizuka, Yoshihito Uchino, Noriyoshi Iriyama, Katsuhiro Miura, Tomohiro Nakayama, Shinobu Masuda, Yoshihiro Hatta, Hideki Nakamura

**Affiliations:** ^1^ Department of Medicine Division of Hematology and Rheumatology Nihon University School of Medicine Tokyo Japan; ^2^ Department of Pathology and Microbiology Division of Laboratory Medicine Nihon University School of Medicine Tokyo Japan; ^3^ Department of Pathology and Microbiology Division of Oncologic Pathology Nihon University School of Medicine Tokyo Japan

**Keywords:** central nervous system, high‐dose chemotherapy, intravascular large B‐cell lymphoma, methotrexate, programmed death‐ligand 1

## Abstract

Intravascular large B‐cell lymphoma (IVLBCL) is a rare subtype of B‐cell lymphoma characterized by aggressive disease progression with a high incidence of central nervous system (CNS) involvement. We retrospectively analyzed 16 patients with de novo IVLBCL treated at our hospital between 2004 and 2018 with either standard therapy plus CNS‐directed therapy or standard therapy alone. CNS‐directed therapy was associated with a significantly better 2‐year CNS‐free survival (100% vs. 63%, *p* = 0.0191), despite no significant effects on progression‐free or overall survival. Further studies should assess CNS‐focused treatment in patients with IVLBCL with or without primary CNS involvement.

## 1 INTRODUCTION

Intravascular large B‐cell lymphoma (IVLBCL) is an extremely rare subtype of B‐cell non‐Hodgkin lymphoma. It is characterized by unique selective disease progression within the lumen of multiple small vessels. Patients experience non‐specific symptoms, such as fever of unknown origin, and have aggressive clinical course, with a tendency toward central nervous system (CNS) invasion and poor prognosis [1]. While IVLBCL management remains challenging, recent studies have provided valuable information for increasing our understanding.

Although there have been no widely accepted prognostic factors for IVLBCL, poor performance status has been identified as one possible factor [2]. Furthermore, expression of programmed death‐ligand 1 (PD‐L1) in tumor cells, which may promote escape from the host's immune system, has been proposed as an adverse prognostic factor in patients with IVLBCL [3]. Regarding the treatment of IVLBCL, integrating CNS prophylaxis using high‐dose methotrexate (MTX) into standard rituximab, cyclophosphamide, doxorubicin, vincristine, and prednisone (R‐CHOP) therapy is expected to improve clinical outcomes [4]. In light of these advances in understanding and treatment of IVLBCL, we aimed to evaluate how CNS‐directed treatment, such as high‐dose MTX, impacts clinical outcomes in real‐world patients with IVLBCL beyond the administration of conventional R‐CHOP.

## 2 METHODS

We retrospectively analyzed patients with de novo IVLBCL treated at our hospital between 2004 and 2018 after approval by the review board. All patients provided written informed consent to their treatments. Clinical records were collected from the database in our clinic. Diagnosis of IVLBCL was based on histopathological examination of specimens from the skin, bone marrow, or involved organs [5, 6].

Primary CNS involvement was defined as any neurologic symptoms with evidence of CNS lesions on magnetic resonance imaging (MRI) or in the cerebrospinal fluid before treatment. Secondary CNS involvement was defined as the emergence of neurologic symptoms caused by isolated CNS relapse or CNS involvement with systemic disease progression, which were confirmed by MRI or spinal tap. In patients with primary CNS involvement, recurrence of neurologic symptoms confirmed by MRI or spinal tap was defined as secondary CNS involvement.

In our institution, treatment regimens for IVLBCL were determined on a case‐by‐case basis, taking patients’ age, anticipated tolerability to intensive chemotherapies, or presence/absence of CNS involvement into account. Thus, we divided patients into the CNS‐directed therapy and standard therapy groups according to their type of chemotherapy regimen. CNS‐directed therapy included R‐CHOP or dose‐intensified R‐CHOP (R‐D‐CHOP) along with high‐dose MTX in the first remission (8 g/m^2^) [7]; R‐D‐CHOP along with high‐dose chemotherapy with cyclophosphamide, etoposide, and ranimustine followed by autologous hematopoietic stem cell transplantation (CEM/ASCT) in the first remission [7, 8]; R‐CHOP along with high‐dose MTX (3.5 g/m^2^ on day 1) and cytarabine (2 g/m^2^ twice a day on days 2‐3) (HDMA) [9]; and rituximab, hyper‐fractionated cyclophosphamide, vincristine, doxorubicin, and dexamethasone alternating rituximab with high‐dose MTX (1.0 g/m^2^ on day 1) /cytarabine (3 g/m^2^ twice a day on days 3‐4) (R‐hyper‐CVAD/R‐MA) [10]. The standard therapy was R‐CHOP alone. The treatment group was per‐planned regimens irrespective of the completion of therapy (i.e., intention‐to‐treat) to minimize selection bias. Immunohistochemical analysis was performed on diagnostic samples using antibodies against PD‐L1 (SP142) (30% of the cut‐off value) [3], CD10 (30%), BCL‐6 (30%), multiple myeloma oncogene 1 (30%) [11], MYC (40%), and B‐cell lymphoma 2 (BCL‐2) (50%) [12, 13]. In situ hybridizations for Epstein‐Barr virus‐encoded small RNA and *MYC* rearrangement were also performed. We estimated 2‐year CNS‐free survival, progression‐free survival (PFS), and overall survival (OS) using the Kaplan‐Meier method in each group to assess the prognostic impact of intensive treatment regimens. Then, we compared survival curves between the groups using the log‐rank test. CNS‐free survival was defined as the period from IVLBCL diagnosis to the date of secondary CNS involvement, progression in non‐CNS lesions (censored), last follow‐up (censored), or death. We used JMP version 14.2 software (SAS Institute Inc.) for statistical analysis. A *p*‐value of < 0.05 was considered statistically significant.

## 3 RESULTS

**FIGURE 1:**
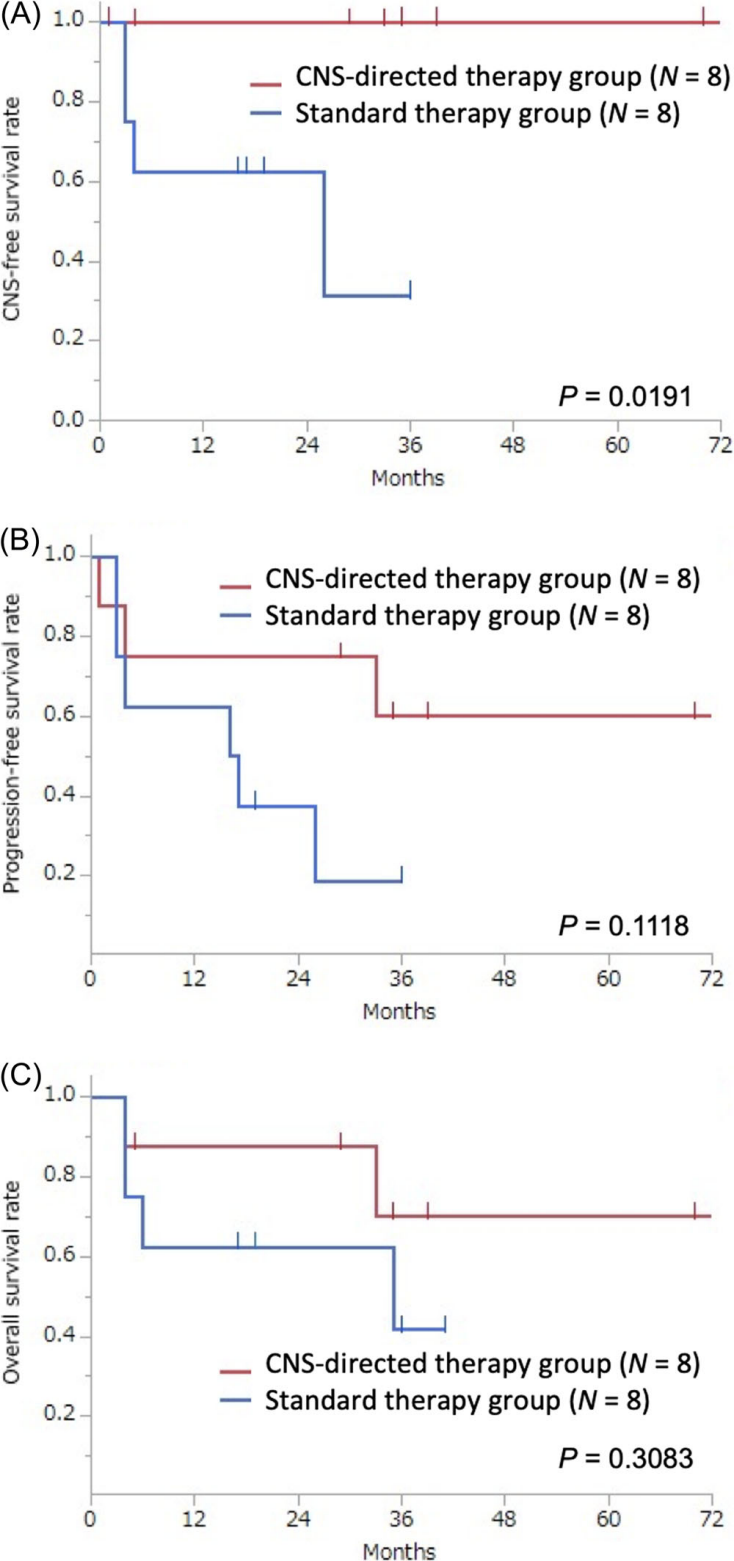
Kaplan‐Meier curves of the central nervous system (CNS)‐free survival rates (A), progression‐free survival rates (B), and overall survival rates (C) according to the type of planned treatment regimens (CNS‐directed therapy plus rituximab, cyclophosphamide, doxorubicin, vincristine, and prednisone [R‐CHOP] [e.g., high‐dose chemotherapy with R‐CHOP‐based therapy] vs. standard R‐CHOP therapy alone)

During the study period, 17 patients were diagnosed with IVLBCL in our institution. All patients were treated with rituximab‐containing immunochemotherapy. Out of these 17 patients, one patient was excluded due to poor clinical records. Data of 16 patients with a median age of 65 (43–79) years were included in the analysis. Planned treatment regimens in the CNS‐directed therapy group were R‐CHOP followed by high‐dose MTX (*n* = 1),1 R‐D‐CHOP followed by CEM/ASCT (*n* = 2), R‐D‐CHOP followed by high‐dose MTX (*n* = 1), R‐CHOP plus HDMA (*n* = 3), and R‐hyper‐CVAD/R‐MA (*n* = 1). Out of these, two patients did not actually receive CNS‐directed chemotherapies due to disease progression. The standard therapy group received R‐CHOP alone (*n* = 8). All treatments were given with curative intent, and regular doses of cytotoxic drugs were administered. Table 1 shows the clinical characteristics, immunohistochemical findings, and planned treatments in all patients. Except for age (median 58 vs. 71 years, *p *= 0.0206), there was no significant difference in these categories between the CNS‐directed and standard therapy groups. Specifically, incidences of any CNS symptoms and primary CNS involvement were not significantly different (50% vs. 25%, *p* = 0.2982 and 25% vs. 25%, *p* = 1.0000, respectively). The completion rates of planned treatments and the complete response rates after initial treatments in these groups were also similar (75% vs. 75%, *p* = 1.0000 and 75% vs. 63%, *p* = 0.6887, respectively). Regarding survival analyses, 2‐year CNS‐free survival was significantly better in the CNS‐directed therapy group than in the standard therapy group (100% vs. 63%, *p *= 0.0191). However, the differences in 2‐year PFS and OS between the two groups did not reach statistical significance (75% vs. 38%, *p *= 0.1118 and 88% vs. 63%, *p *= 0.3083, respectively) (Figure 1).

**TABLE 1:** Patient characteristics

**Categories**	**All patients (*N* = 16)**
Median age, year	65 (43–79)
Sex (male vs female), *n*	7 versus 9
Stage III/IV, *n* (%)	15 (94)
ECOG PS ≥2, *n* (%)	15 (94)
Serum LDH ≥ normal, *n* (%)	15 (94)
Extranodal sites ≥2, *n* (%)	6 (38)
IPI score ≥4, *n* (%)	15 (94)
Any CNS symptoms, *n* (%)	6 (38)
Primary CNS involvement, *n* (%)	4 (25)
HPS subtype, *n* (%)	10 (63)
COO subtypes (GCB vs non‐GC)^a^, *n*	1 vs 15
PD‐L1 ≥30%, *n* (%)	8 of 13, (62)
Double‐expressor lymphoma, *n* (%)	8 (50)
*MYC* rearrangement^b^, *n*	0
EBER positivity, *n*	0
Planned treatment	
R‐CHOP + HD‐MTX	1
R‐D‐CHOP + CEM/ASCT	2
R‐D‐CHOP + HD‐MTX	1
R‐CHOP + HDMA	3
R‐Hyper‐CVAD + R‐MA	1
R‐CHOP alone	8

Abbreviations: BCL‐2, B‐cell lymphoma 2; CEM/ASCT, high‐dose chemotherapy with cyclophosphamide, etoposide, and ranimustine followed by autologous hematopoietic stem cell transplantation; CNS, central nervous system; COO, cell‐of‐origin; EBAR, Epstein‐Barr virus early RNA; ECOG PS, Eastern Cooperative Oncology Group performance status; GCB, germinal center B‐cell like; HDMA, high‐dose MTX and cytarabine; HD‐MTX, high‐dose methotrexate; HPS, haemophagocytic syndrome; IPI International Prognostic Index; LDH, lactate dehydrogenase; NUL, normal upper limit; PD‐L1, programmed death‐ligand 1; R‐CHOP, rituximab, cyclophosphamide, doxorubicin, vincristine, and prednisone; R‐D‐CHOP, dose‐intensified R‐CHOP; R‐Hyper‐CVAD, rituximab, hyper‐fractionated cyclophosphamide, vincristine, doxorubicin, and dexamethasone; R‐MA, rituximab and high‐dose MTX/cytarabine.

^a^ The cell‐of‐origin is determined according to Hans's algorithm.[11].

^b^ Vysis LSI MYC Dual Color Break Apart Rearrangement Probe (Abbott Laboratories, Chicago, IL) is used.

## 4 DISCUSSION

Although our findings were based on a limited number of patients, the present study provides crucial insights into the effects of CNS‐directed chemotherapies alongside standard R‐CHOP. These findings could even benefit patients with IVLBCL with primary CNS involvement. Of note, a pivotal phase‐2 study in previously untreated patients with IVLBCL, in which 38 patients were treated with R‐CHOP alongside high‐dose MTX and intrathecal chemotherapy as CNS‐oriented therapy, excluded patients with primary CNS involvement [4]. That study demonstrated a 76% of 2‐year PFS with a 3% of 2‐year secondary CNS involvement rate [4]. Contrarily, in our study, a substantial number of patients exhibited CNS symptoms or apparent primary CNS involvement in both treatment groups. However, patients treated with CNS‐directed treatment demonstrated better CNS‐free survival and relatively favorable PFS and OS. Generally, approximately one‐quarter of patients with IVLBCL have CNS lesions at initial diagnosis [14]. Thus, the utilities of high‐dose chemotherapies in such patients are rather a subject of debate in the management of IVLBCL.

Given the nature of retrospective studies, confounding factors such as patient age were inevitable in the present study. In addition, treatment regimens in the CNS‐directed treatment group were not uniform. Still, complete remission and treatment completion rates between the groups were not significantly different, which might have mitigated these biases. Furthermore, we carefully evaluated the histopathological features of each group. For instance, Suzuki et al. reported that 12 (35%) of 34 IVLBCL cases exhibited PD‐L1 positivity (PD‐L1^+^) and that the PL‐L1^+^ group showed worse OS compared with the PD‐L1^−^ group [3]. In addition, Boonsakan et al. reported that six (40%) of 15 patients with IVLBCL had the concurrent expression of MYC and BCL‐2 in lymphoma cells (double‐expressor lymphoma). Moreover, these patients with double‐expressor lymphoma showed a higher mortality rate than non‐double‐expressors [15]. These pathologic characteristics between the groups were also balanced in the present study.

In conclusion, our study results may represent a basis for further evaluation of CNS‐focused treatment along with standard R‐CHOP in patients with IVLBCL with or without primary CNS involvement. Therefore, prospective, multicentre studies of this patient population are needed.

## CONFLICT OF INTEREST

Hiromichi Takahashi received honoraria from Bristol‐Myers Squibb. Takashi Hamada received honoraria from Bristol‐Myers Squibb. Masaru Nakagawa received honoraria from Bristol‐Myers Squibb. Noriyoshi Iriyama received honoraria from Novartis, Bristol‐Myers Squibb, Takeda, and Ono Parma. Katsuhiro Miura received honoraria from AstraZeneca, Chugai, Kyowa Kirin, Takeda, Bristol‐Myers Squibb, Nippon Shinyaku, SymBio, and Ono Parma. Yoshihiro Hatta received honoraria from Chugai, Kyowa Kirin, Janssen, Bristol‐Myers Squibb, Takeda, and Ono Parma. Hideki Nakamura received research grants from MSD, Asahi Kasei Pharma, Astellas, AbbVie, Japan Blood Products organization, Eisai, Otsuka Pharmaceutical, Ono Pharma, Kyowa Kirin, Sanofi, Shionogi, Daiichi Sankyo, Taiho, Takeda, Mitsubishi Tanabe, Chugai, Teijin Pharma, Eli Lilly, Nippon Kayaku, Nihon Pharmaceutical, Boehringer Ingelheim, and Pfizer. The other authors have no conflicts of interest to disclose.

## FUNDING INFORMATION

Any third parties did not financially support this study.

## AUTHOR CONTRIBUTIONS

Hiromichi Takahashi, Haruna Nishimaki, Yoko Nakanishi performed the research. Haruna Nishimaki, Yoko Nakanishi, and Shinobu Masuda diagnosed the patients and performed pathological work. Hiromichi Takahashi, Takashi Hamada, Masaru Nakagawa, Kazuhide Iizuka, Yoshihito Uchino, Noriyoshi Iriyama, Katsuhiro Miura, Yoshihiro Hatta, and Haruna Nishimaki treated patients and assembled the data. Hiromichi Takahashi and Katsuhiro Miura designed the research and analyzed the data. Tomohiro Nakayama, Shinobu Masuda, Yoshihiro Hatta, and Hideki Nakamura supervised the study. Katsuhiro Miura wrote the original draft, and all authors critically revised the article and approved the final version to be published.
